# Cross-species referential signalling events in domestic dogs (*Canis familiaris*)

**DOI:** 10.1007/s10071-018-1181-3

**Published:** 2018-04-30

**Authors:** Hannah K. Worsley, Sean J. O’Hara

**Affiliations:** University of Salford, School of Environment & Life Sciences, Peel Building, Salford, Greater Manchester, M5 4WT UK

**Keywords:** Domestic dog, Cognition, Referential gestures, Communication, Cross-species

## Abstract

Referential gestures are used by a signaller to draw a recipient’s attention to a specific object, individual or event in the environment. These gestures have received much research attention in relation to human and non-human primates with great apes being shown to possess impressive gestural repertoires. Domestic dogs (*Canis familiaris*) meanwhile provide an ideal non-primate candidate for investigating referential signalling due to their unique relationship with humans that centres on non-verbal communication with frequent interaction. Here we observed 37 pet dogs in their own homes. Owners recorded 242 videos containing 47 potential referential gesture events. We analysed those recordings to reveal evidence of 19 referential gestures performed by domestic dogs during everyday communicative bouts with humans, showing that the gestures conform to the five features of referential signalling. Our study exposes impressive gesturing abilities in a non-primate mammal; especially when viewed in the context of the cross-species rather than intraspecific communication.

**Electronic supplementary material:**

The online version of this article (10.1007/s10071-018-1181-3) contains supplementary material, which is available to authorized users.

## Introduction

Referential gestures are produced to direct attention (Leavens 2004). They are mechanically ineffective movements of the body which are repeated and elaborated on until they elicit a specific response from an intended recipient (Bates et al. 1975; Hobaiter and Byrne 2011; Malavasi and Huber 2016; Warneken et al. 2006). From an early age, human infants use gestures to draw a recipient’s attention to objects they desire (Bates 1979) and it has been suggested that most communicative events contain both motivational and referential components (Hauser 1996; Marler et al. 1992). Pointing is the most commonly used human referential gesture (Liszkowski et al. 2012) and is thought to be a key component of human language development (Franco and Butterworth 1996), as it strongly predicts language acquisition (Bates 1979; Colonnesi et al. 2010).

For a gesture to be considered as referential in function it must conform to five features. First, it must be directed toward an object or specific area of the signaller’s body, e.g., a child pointing towards a specific toy. Second, it is a mechanically ineffective movement, e.g., a gesture that is not designed to act as a direct physical agent such as the human pointing gesture. Third, it is aimed at a potential recipient and fourth, receives a voluntary response from that recipient, e.g., a child repeatedly points at a toy and then looks at/points at their mother who then, of her own accord, retrieves the toy and gives it to the child. Finally, a referential gesture must also demonstrate hallmarks of intentional production, e.g., a child repeatedly points at a toy then waits for a response from their mother; when no response is forthcoming the child continues to point at the toy but also introduces a new gesture, such as grabbing air, so as to achieve their goal (Pika and Bugnyar 2011; Vail et al. 2013).

Referential gestures are non-accidental. Therefore, a signaller needs to demonstrate an intention to communicate with their intended recipient (Savilli et al. 2016). There are five attributes of intentional communication (Genty et al. 2009) in contemporary use. For a gesture to be considered intentional it must be given by an individual in a goal-directed way (Genty et al. 2009). The obtaining of a result provided the motivation for producing a gesture and the recipient’s actions must satisfy the signaller to indicate their intentions (Hobaiter and Byrne 2014). If the outcome is not satisfactory to the signaller, response waiting is expected to be seen (Call and Tomasello 2007; Tomasello et al. 1994), followed by repetition of the gesture or incorporation of others in a process referred to as persistence and elaboration (Cartmill and Byrne 2007; Leavens et al. 2005). A final criterion for an intentional gesture is that it must be directed at an audience (Genty et al. 2009). According to Malavasi and Huber (2016), to be considered as referential a gesture must show at least some of these attributes of intentionality; in particular persistence and elaboration (Woodruff and Premack 1979).

In contrast to their frequent use by humans, referential gestures in non-human taxa are relatively rare (Vail et al. 2013). Most research demonstrates the use of referential gestures by great apes in captivity, where subjects gesture to a human experimenter (Cartmill and Byrne 2007; Leavens et al. 2004, 2005; Leavens and Hopkins 1998; Woodruff and Premack 1979). In the wild, chimpanzees (*Pan troglodytes*) use a vocalisation known as the ‘rough grunt’ (Goodall 1986) as a referent in feeding contexts (Slocombe and Zuberbühler 2005) and will use directed scratches potentially indicating an area of the body they wish the recipient to groom (Pika and Mitani 2006).

Referential gesturing, however, is not unique to primates. Ravens (*Corvus corax*), for cexample, have been observed performing object-orientated behaviours to direct the attention of their conspecifics (Pika and Bugnyar 2011). Moreover, some species of coral reef fishes, the grouper (*Plectropomus pessuliferus marisrubri*) and coral trout (*Plectropomus leopardus*), use referential gestures to indicate the location of hidden prey (Vail et al. 2013). Interestingly, Vail et al. (2013) also reported that groupers and coral trout use these referential signals to initiate cooperation with hunting partners.

Companion domestic dogs present an interesting case for the study of referential gestures as they spend most of their time interacting and communicating with heterospecifics. Investigations into dog–human communication have revealed that interactions between humans and dogs have referential components (Bensky et al. 2013). Dogs have a set of skills that allow them to use and understand human-produced referential cues (Agnetta et al. 2000), even out-performing other domesticated animals in these tasks (McKinley and Sambrook 2000).

Domestic dogs can also perform ‘showing’ behaviours in referential communicative bouts. ‘Showing’ behaviours are defined as communication which contains both a directional element related to an external object and an attention-getting element that directs the attention of the recipient to the signaller (Miklósi et al. 2000). Investigators have demonstrated that dogs use the position of their body to indicate the location of a goal object (Gaunet and Deputte 2011) and alternate their gaze between an object of apparent interest and the human while barking (Miklósi et al. 2000), thereby communicating their intentions.

Thus far, dog–human communicative research has tended to focus on dogs’ ability to understand human-given gestures. Research has shown us that from a very early age (6 weeks) puppies can follow a human pointing gesture (Hare et al. 2002; Riedel et al. 2008). When completing an object choice test (locating hidden food using a social cue) dogs understand several different human-given social cues: (a) a human pointing at the food location; (b) a human orientating their gaze to the target location; and (c) a human bowing or nodding at the target location (Hare et al. 1998; Miklósi et al. 1998). Dogs also perform at above chance levels when using social cues produced by unfamiliar humans and conspecifics (Hare and Tomasello 1999) and are successful in following the pointing gesture given by an artificial hand (Kundey et al. 2014). Knowledge concerning dogs’ abilities to produce gestures that can be understood by humans, by contrast, is lacking. Here we attempt to bridge that gap by observing gestures that pet dogs direct to their owners during everyday communicative bouts to investigate referential gesturing and humans’ ability to understand the gestures performed by dogs.

## Materials and methods

### Subjects

We recruited the owners of 37 domestic dogs (16 female, 21 male, aged 1.5–15 years) who had lived with their owners for a minimum of 5 months before the start of the study. For information about the subjects, breed, sex, age, number of people who live with the dog, where the dog came from, length of time with current owners, number of videos provided by owners and data collection time see Supplementary Material: Table SM1.

### Data collection

To maximise the quantity of data we could accumulate, we used a citizen science method to collect data on the communicative abilities of dogs. This citizen science approach was founded on the method utilised by Horowitz and Hecht (2016) in their ‘play with your dog’ study. In that study, Horowitz and Hecht, asked owners to record themselves playing with their dogs and upload the video clips to a specifically designed website. The researchers then behaviourally coded the video clips to identify the characteristics of everyday dog–human play (Horowitz and Hecht 2016).

In our study, participants were asked to film their dogs performing ‘everyday’ communicative bouts (e.g., requesting food and doors to be opened, playing and requesting to be scratched), using their mobile phone whenever the behaviours occurred. To orientate owners to the *kinds* of things we were looking for them to record, all participants were shown pre-collected footage provided by the researcher to aid in their data collection. There was no limit placed on collection and the same kinds of communicative bouts could be recorded multiple times.

The citizen science approach here is equivalent to all-occurrences sampling used by field biologists and involved the owner(s) performing observations of their dog in their home. Our aim was to employ a procedure somewhat analogous to field studies of primates (and other free-ranging animals). It is important to note that some behaviours may have been missed in some subjects. Citizen science relies on the public collecting the data and here it is highly likely that not all gestures have been documented. Nonetheless, this was an acceptable trade off as we gained access to a large corpus of data whilst embracing an inclusive approach that benefits owners (Hecht and Spicer-Rice 2015) and potentially dogs.

To further increase validity, participants were provided with a help sheet to assist them during the observational period and we provided our contact details in case any help was required. We contacted participants at 2 weeks intervals to ensure data collection was going smoothly.

Video data were transferred onto a supplied USB drive. We analysed the footage, coding it according to the dog’s perceived goal (food, play, etc.). We also asked owners to review their footage and label the dog’s perceived goal, referred to as their apparent satisfactory outcome (ASO). Not all participants completed this part of the study but 97.6% of researcher labels matched the owner labels.

We collected data on the subject’s sex, age, size of household and length of time in the household. In great apes, repertoire size differs as a function of age class in both chimpanzees (Hobaiter and Byrne 2011) and gorillas (*Gorilla gorilla*) (Genty et al. 2009). Sex differences in apes have not been reported, but sex differences in basic cognitive abilities has been reported in domestic dogs (e.g., Müller et al. 2013). Consequently, age and sex could impact the repertoire size of dogs. Furthermore, for domestic dogs an individual’s environment shapes the behaviour they exhibit over their lifetime (Udell and Wynne 2008). Therefore, the number of people that live with the dog and the length of time the dog has lived with those owners each has the potential to impact on repertoire size.

### Analyses

Gestures were categorised as per their apparent satisfactory outcome (ASO). ASOs are deduced from a plausible desire and signaller satisfaction (Hobaiter and Byrne 2014). They produce an outcome that results in the termination of communication. We initially identified eight ASOs, three of which were excluded from further analysis due to low observation frequency (*n* = 7). A further ASO, “Play with me!” was also excluded as some gestures used during play are also used with other meanings in other ASOs (Hobaiter and Byrne 2014). This gave us four ASOs which yielded the highest frequency of observations to decipher potential referential gestures. Gestures were initially identified as discrete, mechanically ineffective actions (*sensu* Genty et al. 2009; Hobaiter and Byrne 2011, 2014). These actions included limb, head and whole body movements but not facial expressions or static body stances (Hobaiter and Byrne 2011, 2014). The five features of referential signalling were then applied to determine the frequency of actual referential gestures observed. Where a portfolio of gestures, each separated by less than 1 s was recorded, we applied the referential criteria to each single gesture within the portfolio (Hobaiter and Byrne 2011, 2014).

### Reliability

Inter-rater reliability analysis using Cohen’s kappa was performed to ascertain consistency between observers on a sample of 60 videos. Sixty videos is equivalent to 25% of the 242 bouts of communication collected for this study. The secondary observer was trained to identify referential gestures using the data from one subject (St.W). Both observers recorded the gesture and the time at which it occurred, then agreements and disagreements between the two observers were scored (Bateman and Gottman 1997). Cohen’s kappa revealed a good agreement between the coders for the number of referential gestures recorded and the times at which they were performed, kappa = 0.642, *p* < 0.0001.

### Statistical analyses

All statistical tests were performed using IBM SPSS Statistics (version 24) with the significance level set at *p* < 0.05. We performed a multiple regression analysis after testing the data met the assumptions of linear regression. We looked at what factors influenced the size of the gestural repertoire using sex (categorical variable) and age, number of people who live with the dog and length of time spent with current owners (continuous variables). Volume of data collected (number of videos) was included as a potential confounding factor as different quantities of data were collected for each subject.

## Results

The four ASOs with the highest observational frequency were “Scratch me!”, “Give me food/drink”, “Open the door” and “Get my toy/bone”, resulting in 242 bouts of communication. Within these 242 bouts we initially identified 47 potential referential gestures (suppl. material: Table SM2) performed by dogs which conformed to all or some of the five features for referentiality (Supplementary Material: Table SM3). Once we applied the five features for referential communication (Table 1) this reduced to 19 gestures having referential properties (Table 2).

**Table 1:** How observed dog gestures conform to the five features of referentiality

Referential criteria	Occurrence (yes/no)	Description of findings
1. Directed towards an object or specific area of the signaller’s body	✓	Most gestures were directed whilst at the location of the apparent goal. However, some were performed away from the goal location with the apparent aim of leading the recipient to the ASO
2. Aimed at a potential recipient	✓	The intended recipient was the individual filming as all gestures were performed to the camera. Therefore, all gestures were apparently aimed at an attending recipient
3. Receive a voluntary response	✓	All gestures when performed individually and within a portfolio prompted a voluntary response from the intended recipient
4. Are mechanically ineffective	✓	All gestures were performed in the presence of a recipient with the apparent aim of recruiting them to attain an ASO. If these gestures could be directly used to achieve an ASO dogs would not look to a potential recipient for support but would be able to obtain the ASOs without assistance
5. Hallmarks of intentional production	✓	Gestures were performed in a goal-directed way with the apparent aim of achieving some plausible desired result (ASOs). Dogs were persistent in their performance of gestures until the apparently desired outcome was achieved and all communication observed was directed to an appropriate audience. Persistence and elaboration of gestures, was exhibited if dogs did not initially achieve the ASO (*n* = 24) and if the receiver was not sufficiently quick to respond (*n* = 218)

**Table 2:** Definitions of the 19 referential gestures observed in cross-species domestic dog communication

Gesture	Definition
Roll over	Rolling onto one side of the body and exposing the chest, stomach and groin
Head under	Plunge headfirst underneath an object or human
Head forward	Move the head forwards and up to direct a human’s appendage to a specific location on the body
Hind leg stand	Lift front paws off the ground and stand on hind legs, front paws are not resting on anything
Head turn	Head is turned from side to side on the horizontal axis usually between a human and an apparent object of interest
Shuffle	Shuffle whole body along the ground in short movements, performed whilst in roll over position
Back leg up	Lifting of a single back leg whilst lay on one side of the body
Paw hover	Hold one paw in mid-air whilst in a sitting position
Crawl under	Move entire or part of body underneath an object or a human’s appendage
Flick toy	Hold toy in the mouth and throw it forwards, usually in the direction of a human
Jump	Jump up and down off the ground, human or an object, usually while staying in one location
Paw reach	Placing a single paw or both paws underneath another object to retrieve an object of apparent interest
Nose	Pressing nose (or face) against an object or human
Lick	Licking an object or human once or repetitively
Front paws on	Lifting both paws off the ground and resting them on an object or human
Paw rest	Lifting a single front paw and resting it on an object or human
Head rub	Involves rubbing the head against an object or human on which the signaller is leaning on
Chomp	Involves opening the mouth and placing it over the arm of a human whilst repeatedly and gently biting down on the arm
Paw	Lifting of a single front paw to briefly touch an object or human

We recorded 1136 instances of the 19 referential gestures from 242 bouts of communication, however, only 1016 of these instances demonstrated hallmarks of intentional production (Table 3). These 120 instances were excluded from the analysis due to not conforming to all five criteria for referentiality.

**Table 3:** Total number of referential gestures observed in each ASO alongside the actual number of gestures which also conformed to the criterion of intentional production

Gesture	1. “Scratch me!”	2. “Give me food/drink”	3. “Open the door”	4. “Get my toy/bone”
Roll over	18 (14)	0	0	0
Head forward	12 (10)	16 (16)	6 (6)	0
Nose	44 (36)	23 (23)	13 (13)	16 (16)
Paw	33 (32)	51 (43)	36 (35)	102 (98)
Paw hover	6 (6)	31 (27)	6 (3)	4 (2)
Head turn	33 (20)	223 (195)	117 (110)	61 (56)
Lick	46 (39)	8 (8)	8 (8)	1 (1)
Head rub	2 (2)	0	0	0
Paw rest	11 (9)	0	3 (2)	0
Hind leg stand	0	5 (4)	1 (1)	2 (2)
Front paws on	12 (11)	22 (17)	28 (26)	4 (4)
Jump	0	16 (11)	19 (17)	2 (1)
Head under	2 (2)	1 (1)	0	54 (54)
Paw reach	0	0	0	21 (20)
Crawl under	0	0	0	2 (2)
Chomp	5 (5)	0	0	0
Shuffle	3 (2)	0	0	0
Back leg up	3 (2)	0	0	0
Flick toy	0	4 (4)	0	0
Total	230 (190)	400 (349)	237 (221)	269 (256)

The “Scratch me!” ASO produced the largest repertoire with 14 referential gestures being recorded. Both the “Give me food/drink” and “Get my toy/bone” ASO produced 11 referential gestures and in the “Open the door” ASO 10 referential gestures were observed. All 37 subjects were observed using referential gestures in at least one of the four ASOs but not all dogs performed the same gestures and there was variation between dogs in the repertoire size for each ASO (Supplementary material: Table SM4). Some gestures were used by dogs for more than one ASO, in different contexts.

Individual gestural repertoire was shown to increase with both number of people who live with the dog and the number of videos collected (Table 4). Sex, age and amount of time dogs spent with current owners were found to be not significant predictors of repertoire size.

**Table 4:** Regression output showing the variables which do and do not have an effect on the size of an individual dogs’ gestural repertoire

Model	Unstandardized coefficients		Standardized coefficients		Sig	95% confidence interval for *B*	
	*B*	SE	Beta	*t*		Lower bound	Upper bound
Coefficients							
Constant	2.520	1.709		1.475	0.150	− 0.965	6.006
n_people	0.850	0.407	0.296	2.088	0.045	0.020	1.680
Sex	− 1.169	0.735	− 0.213	− 1.589	0.122	− 2.668	0.331
Age	− 0.060	0.463	− 0.070	− 0.130	0.897	− 1.004	0.884
n_videos	0.168	0.049	0.504	3.448	0.002	0.069	0.267
n_time	0.113	0.444	0.136	0.254	0.801	− 0.793	1.018

**Fig. 1:**
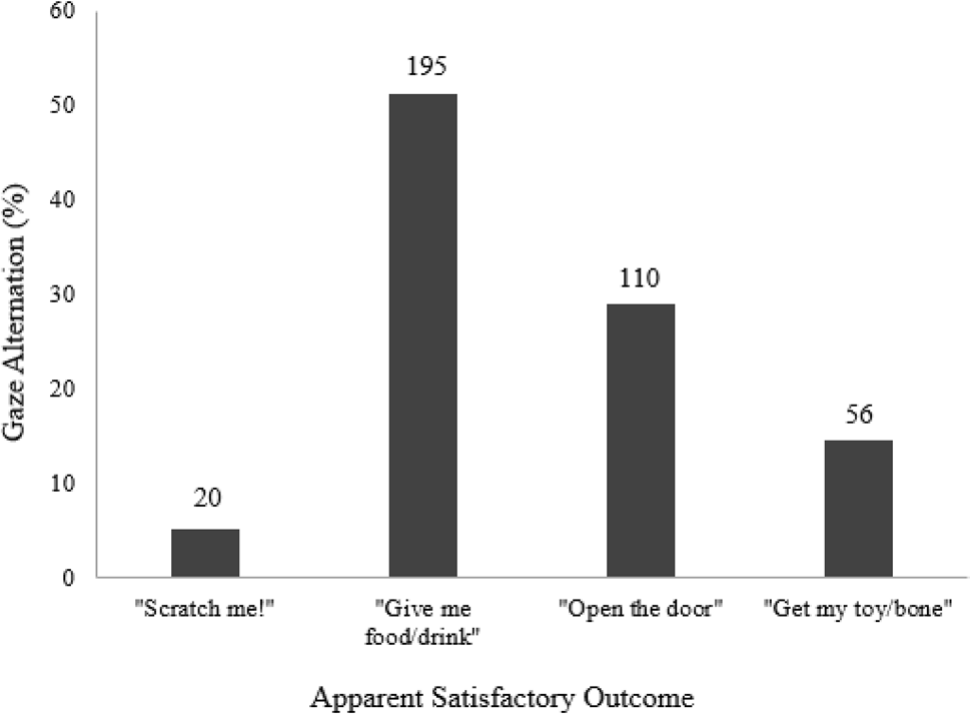
Percentage of gaze alternation gestures observed in each ASO with actual number above each bar

The most common gesture observed involved gaze alternation (head turn) gestures, recorded 381 times over all four ASOs (Fig. 1). Thirty-five of the 37 dogs were observed to use the head turn gesture.

## Discussion

This study provides strong evidence that pet dogs use referential gestures during everyday communicative bouts with humans. Gestures were performed in a referential way, with the attention of the receiver drawn to an item that was of apparent interest to the signaller. Furthermore, our results show that humans responded to these signals in ways that apparently satisfied the signaller. Kaminski et al. (2011) showed that dogs will gesture towards an object more frequently when it is something of apparent interest to them. Consistent with that assertion, the ASOs identified here all involved an outcome which benefited the dog and not the owner.

Evidence of referential communication in great apes has primarily consisted of pointing gestures performed by captive chimpanzees (Leavens et al. 1996) and orangutans (*Pongo pygmaeus*) (Call and Tomasello 1994), and wild bonobos (*Pan paniscus*) (Douglas and Moscovice 2015) and chimpanzees (Hobaiter et al. 2014); although there is some evidence of wild chimpanzees performing ‘directed scratches’ gestures to request grooming of specific areas of the body (Pika and Mitani 2006). It is further reported that one species of monkey, the bonnet macaque (*Macaca radiata*), uses four distinct intentional referential gestures (position change, head/body extension, showing rear, holding body part) during allogrooming (Gupta and Sinha 2016). Dogs lack the comparable anatomy to easily perform similar overt pointing gestures; however, we did find evidence of dogs directing owners to areas of the body in the “*Scratch me!*” ASO (Roll over, Head forward, Back leg up).

We also revealed high occurrences of gaze alternation (Head Turn) in dogs which, moreover, was not limited to one ASO. In the majority of cases (96.1%, *n* = 366) of gaze alternation identified in the study, dogs were initially looking at the agent, then switched their gaze toward the apparent goal before turning back to look at the receiver again. Gaze alternation is viewed as one of the best means of referential gesturing (Akhtar and Gernsbacher 2008) with pre-verbal human infants (Leavens et al. 1996) and great apes (Leavens et al. 2004, 2005; Leavens and Hopkins 1998) regularly performing it. The occurrence of the gaze alternation gesture suggests that dogs are potentially adept at using referential communication.

Our study identifies an impressive 19 referential signals in domestic dogs. It is important to note that training may have had an effect on individual dogs’ referential repertoire. For example, a dog that has been trained to not jump is less likely to use that gesture as a referent when compared to another dog in which the behaviour has not been extinguished through training. Our results also revealed that dogs call upon a portfolio of referential gestures to indicate a single reward. This could have been due to the delay in recipient response created by filming, but it demonstrates that dogs can elaborate on their initial gesture when an appropriate response from the recipient has not been elicited. This suggests that dogs possess repertoire flexibility and are able to still communicate effectively with their owners even when specific behaviours have been expunged through training.

Udell and Wynne (2008) have suggested that a dog’s environmental history has a major effect on the shaping of behaviour, and interestingly our results revealed that the size of an individual’s referential gestural repertoire is directly proportional to the number of people who live with the dog. The inference being that dogs with a larger number of people to communicate with possess a greater number of gestures to call upon since they have had more opportunities to learn, and thus increase their repertoire size. This implies dog gestures are not recipient-dependent but that they are performing their portfolio of gestures to their human social partners, ensuring they are understood by the recipient. Our results also revealed a direct relationship between gestural repertoire size and the number of videos collected such that repertoire size increased as more data were collected. This is an expected outcome of our data collection procedure, with varying amounts of data collected across participants. It does, however, inform us that our overall estimate of the size of dogs’ referential gestural repertoire (*n* = 19) is likely to be a conservative estimate. Future investigation is likely to lead to the discovery of new gestures in this species.

We further found no effect of age (or sex) on repertoire size in dogs. This is in contrast to findings in great apes where repertoire size is negatively related to age (Genty et al. 2009; Hobaiter and Byrne 2011). There it is proposed apes gradually learn which gestures from a portfolio work best and so omit superfluous ones with experience (Byrne et al. 2017). With the so-called ‘redundancy’ taking place adult apes consequently demonstrate fewer gestures. This refinement learning appears to not be evident in dogs who instead continue to throw all gestures at the target individual perhaps in the hope that one will be understood. Longitudinal studies on gestural ontogeny, however, are required to confirm this.

The prevalence of referential communication in dogs suggests that the ability is not as rare as previously thought (Veà and Sabater-Pi 1998) but could be a common aspect of dog–human communication. Dogs can interpret and understand human-given referential gestures with ease (Kaminski and Nitzschner 2013) and the evidence from our study suggests humans are also able to successfully interpret and understand canine-given referential gestures. From the age of 5 weeks, puppies look more toward humans than conspecifics (Gácsi et al. 2005) indicating that the ability to communicate with humans emerges at a very early age. This suggests that the co-habitation process may have resulted in a change in the cross-species communicative abilities of both humans and dogs which may explain how both have become skilled at identifying and understanding each other’s referential cues.

To date the majority of canine referential research has investigated dogs’ abilities in response to human-given referential gestures. The current study is one of the first to record and analyse the referential communicative repertoire of domestic dogs during cross-species interactions with humans. The majority of non-canine referential gestural research has concerned itself with subjects who all gesture to conspecifics. The current study has shown that dogs (and humans) are doing something remarkable, having had a shared existence for only 30,000 years (Miklósi 2007). Despite the brevity of this shared existence, dogs have developed a strong relationship with their human social partners (Berns et al. 2015; Hare and Tomasello 2005; Miklósi 2007), with inter-dependence facilitating successful cross-species communication.

The ability to successfully communicate cross-species is theoretically more cognitively challenging than intraspecific communication since it requires an individual to adjust its behaviours so that the other species is able to understand and correctly respond to them. The inference from great ape studies is that the increased ‘intelligence’ in their subjects is due to phylogeny and a shared ancestry with humans (Hobaiter and Byrne 2011). In contrast dogs last shared a common ancestor with primates 100 mya yet this study suggests they possess impressive skills in this domain.

## Electronic supplementary material

Below is the link to the electronic supplementary material.

Supplementary material 1 (DOCX 44 KB)
