# Supplementary material for: Cross-species referential signalling events in domestic dogs (Canis familiaris)
Source: Anim Cogn. 2018 Apr 30;21(4):457–65. doi: 10.1007/s10071-018-1181-3 (PMC6004278; doi:10.1007/s10071-018-1181-3)
Supplement: Supplementary file 1 — Supplementary material 1 (DOCX 44 KB) [file 10071_2018_1181_MOESM1_ESM.docx]

**Cross-Species Referential Signalling Events in Domestic Dogs (*Canis familiaris*)**

*Animal Cognition*

Hannah K. Worsley and Sean J. O’Hara

University of Salford, School of Environment & Life Sciences, Peel Building, Salford, Greater Manchester, M5 4WT

Corresponding author email: h.k.worsley@edu.salford.ac.uk

**SUPPLEMENTARY MATERIALS**

*SM1. Information about the canine subjects, number of videos provided by owners and data collection time*

| Subject ID | Sex | Age (Y) | Breed | People who live with dog (*N*) | Where the dog came from | Length of time with current owners | Videos  (*N*) | Data collection time (weeks) |
| --- | --- | --- | --- | --- | --- | --- | --- | --- |
| Pu.S | F | 2 | Pug | 2 | Breeder | 2 year | 4 | 6 |
| To.S | M | 4.5 | Shihtzu X Toy Poodle | 2 | Breeder | 4 years | 10 | 6 |
| Ma.W | F | 4 | Yorkiepoo | 4 | Kennel | 4 years | 12 | 2 |
| Sh.L | M | 3 | Miniature Poodle X Chinese Crested Powder Puff | 4 | Breeder | 3 years | 13 | 4 |
| Wa.L | M | 3 | Miniature Poodle X Chinese Crested Powder Puff | 4 | Breeder | 3 years | 15 | 4 |
| St.W | M | 15 | Patterdale Terrier | 4 | Breeder | 15 years | 52 | 2 |
| Ma.Wd | M | 5 | Short Legged Jack Russell | 3 | Adopted from a family member | 3 years | 13 | 4 |
| Su.W | F | 11 | Labrador | 4 | Breeder | 10 years | 9 | 8 |
| Pa.B | M | 4.5 | Jack Russell X | 3 | Farm | 4.5 years | 21 | 2 |
| Em.P | F | 5 | Saint Bernard | 2 | Breeder | 5 years | 8 | 4 |
| Ti.D | F | 8 | Border Terrier | 3 | Breeder | 8 years | 9 | 6 |
| Te.D | F | 8 | Border Terrier | 3 | Breeder | 8 years | 9 | 6 |
| De.F | M | 4 | West Highland Terrier | 3 | Breeder | 3.5 years | 8 | 4 |
| Lo.S | F | 7 | Border Terrier | 3 | Breeder | 7 years | 13 | 4 |
| Ma.B | M | 9 | Jack Russell X Border Terrier | 4 | RSPCA | 8.5 years | 14 | 8 |
| Ph.J | F | 1 | Cockerpoo | 4 | Breeder | 5 months | 17 | 4 |
| Fl.H | F | 7 | English Cocker Spaniel | 4 | Breeder | 7 years | 15 | 4 |
| Os.B | M | 7 | Cocker Spaniel | 4 | Breeder | 7 years | 11 | 6 |
| Ky.H | M | 5 | Beagle | 2 | Breeder | 5 years | 14 | 6 |
| Pe.S | F | 5 | Border Terrier | 3 | Breeder | 5 years | 12 | 4 |
| Mi.C | M | 3 | Border Collie X | 1 | Gumtree Advert | 2.5 years | 15 | 4 |
| Aa.S | M | 7 | Mixed Breed | 2 | Breeder | 7 years | 5 | 8 |
| Ru.S | M | 4 | German Pointer X French Spaniel | 5 | Breeder | 3 years | 20 | 8 |
| Iz.W | F | 5 | Cavalier King Charles Spaniel | 2 | Breeder | 5 years | 5 | 6 |
| Ly.W | F | 6 | Cavalier King Charles Spaniel | 2 | Internet Advert | 2.5 years | 9 | 6 |
| Le.T | M | 9 | Staffordshire Bull Terrier | 2 | Adopted from a family member | 7 years | 4 | 4 |
| Yo.B | F | 2 | Jack Russell X Lhasa Apso | 4 | Rehomed via Internet advert | 21 months | 15 | 6 |
| Bo.H | M | 12 | West Highland Terrier | 4 | Breeder | 12 years | 7 | 4 |
| Du.L | M | 2 | Golden Retriever | 2 | Breeder | 2 years | 6 | 2 |
| Bo.L | M | 9 | German Shepherd | 2 | Breeder | 9 years | 3 | 2 |
| Ar.H | M | 1.5 | Cairn Terrier | 3 | Breeder | 1.5 years | 8 | 10 |
| Ja.B | M | 2.5 | Cocker Spaniel | 2 | Breeder | 2 years | 22 | 6 |
| Je.S | F | 1 | German Shepherd X Akita | 2 | Re-homed | 11 months | 9 | 6 |
| Je.K | M | 3 | Beagle | 3 | Rescue Centre | 2.5 years | 10 | 6 |
| Ba.K | M | 4 | Border Collie | 3 | Farm | 3.5 years | 18 | 6 |
| On.C | M | 7.5 | Lhasa Apso | 2 | Breeder | 7 years | 10 | 4 |
| Be.C | F | 4 | Lhasa Apso X | 2 | Rescue | 2 years | 12 | 4 |

*SM2. Possible referential gestures recorded during initial observations of the video data*

| Subject ID | Referential Signalling | | | |
| --- | --- | --- | --- | --- |
|  | ***“Scratch me!”*** | ***“Give me Food/Drink”*** | ***“Open the Door”*** | ***“Get my Toy/Bone”*** |
| To.S | Roll Over; Lean back | Paw; Lick lips | Nose; Head turn; Look behind |  |
| Pu.S |  | Head turn |  | Head under; Paw |
| Ma.W | Head forward; Head back | Head turn; Hind leg stand; Lick lips | Head turn |  |
| Sh.L | Nose; Roll over |  | Paw |  |
| Wa.L | Body lean; Roll over | Head forward; Head turn; Lick lips | Head turn; Head forward |  |
| St.W | Roll over; Shuffle | Head turn; Head up; Nose; Paw; Lick lips; Stamp paws | Head turn; Head up; Nose; Head forward; Look behind | Head under; Paw reach; Head down |
| Ma.Wd | Body lean; Nose press; Paw; Head forward | Head turn; Head up | Head up; Look behind; Spin bounce |  |
| Su.W | Roll over; Head forward; Lean forward; Back leg up; Lean back; Nose | Head up; Head turn; Lick lips |  |  |
| Pa.B | Roll over | Head up; Head turn; Lick lips | Head turn; Look behind; Nose; Front paws on; Jump | Head down; Paw reach; Head under; Nose |
| Em.P | Paw hover; Chin rest | Head up; Head turn; Stamp paws | Head turn; Head up | Head down; Head under; Paw |
| Ti.D |  | Head turn; Head up; Lick lips; Stamp paws | Head up; Head turn |  |
| Te.D |  | Head turn; Lick lips | Head up; Head turn |  |
| De.F | Groaning; Chin rest; Nose | Head turn; Head up; Begging gesture; Hind leg stand; Lick lips | Look behind; Head turn; Head up | Paw reach; Head down; Head turn |
| Lo.S | Head forward; Roll over; Paw; Nose | Paw; Lick; Head up; Head turn; Lick lips; Stamp paws | Side-step; Front paws on; Head turn; Head up; Jump | Head down; Nose; Head under; Head turn |
| Ma.B | Nose; Paw | Head up; Head turn; Lick lips; Stamp paws | Look behind; Head turn; Paw | Head under; Head turn; Head down; Crawl under |
| Ph.J |  | Head up; Head turn; Lick lips | Head up; Head turn; Circle; Front paws on; Paw; Head forward | Paw reach; Head under; Circle; Paw; Nose; Head down |
| Fl.H | Paw; Lick; Nose; Paw rest; Puppy dog | Head turn; Head up; Turn; Stamp paws | Head up; Turn; Head turn; Look behind; Paw | Head down; Head under; Head turn; Nose |
| Os.B |  | Grab toy; Flick toy; Head up; Hind leg stand; Head turn; Lick lips; Stamp paws | Head turn; Paw | Head down; Head turn; Head under; Paw reach |
| Ky.H |  | Head up; Head turn; Stamp paws; Chin rest; Rest & wait; Lick lips |  | Head under; Nose; Paw reach; Paw; Head down; Head turn |
| Pe.S |  | Head up; Head turn; Stamp paws; Lick lips | Head turn; Head up; Look behind | Head turn; Head down; Head under |
| Mi.C | Paw rest; Paw; Nose; Lick; Paw hover | Lick; Lick lips; Head turn; Stamp paws; Chin rest | Look behind; Head turn; Head up; Front paws on; Paw; Nose |  |
| Aa.S |  | Head turn; Head up; Stamp paws |  |  |
| Ru.S |  | Head turn; Lick lips; Head up; Stamp paws; Paw; Chin rest; Rest & wait; Drop toy; Nose; Circle |  | Head down; Head under; Paw; Nose;  Paw reach |
| Iz.W |  | Head turn; Head up; Lick lips; Paw; Nose | Toy in mouth; Head turn |  |
| Ly.W | Front paws on;  Lick; | Stamp paws; Head turn; Head up; Lick lips; Hind leg stand; Lick | Head turn; Head up |  |
| Le.T |  | Head turn; Head forward; Front paws on; Lick lips | Head up; Head turn; Look behind |  |
| Yo.B | Front paws on; Lick; Nose; Roll over; Head turn;  Head forward;  Paw hover | Head up; Head turn; Chin rest; Rest & wait | Head up; Head turn; Circle |  |
| Bo.H |  | Head up; Stamp paws; Head turn; Lick lips; Paw hover | Paw; Nose; Look behind; Head turn; Head up | Head down; Paw; Head turn |
| Du.L | Chin rest |  | Head up; Head turn; Look behind |  |
| Bo.L |  | Nose; Lick lips; Head up; Head turn |  |  |
| Ar.H |  |  |  | Head under;  Head down |
| Ja.B |  | Stamp paws;  Head turn; Head up | Head turn; Nose; Chin rest; Rest & wait; Head up;  Look behind; Rub head; Down-Up | Head under; Head turn; Nose; Head down; Paw |
| Je.S | Lick; Chomp; Paw; Paw rest; Nose;  Head turn |  | Front paws on; Knock on door;  Head turn; Head up; Look behind |  |
| Je.K | Front paws on; Paw hover; Paw; Paw rest | Head turn; Lick lips; Paw hover;  Hover & wait;  Paw; Stamp paws; Look behind; Head up |  |  |
| Ba.K |  | Head turn; Sway |  |  |
| On.C | Head rest; Head rub | Head up; Head turn; Paw shove; Lick lips; Stamp paws; Turn; Paw;  Front paws on | Look behind;  Head up; Paw; Nose |  |
| Be.C | Head turn; Roll over | Head up; Head turn; Stamp paws; Lick lips;  Paw; Lick | Look behind;  Head turn; Head up |  |

*“Scratch me!”*: 22 potential referential gestures

“*Give me food/drink”*: 24 potential referential gestures

“*Open the door”*: 18 potential referential gestures

*“Get my toy/bone”*: 8 potential referential gestures

Of the 47 gestures initially identified from the video footage (*Table SM2*), 28 failed to conform to all five features of referentiality. Although those gestures were performed intentionally and with the aim of attracting a potential recipient they did not direct the recipient’s attention towards a desired goal.

*SM3. Potential referential gestures initially identified against the strict criteria for referentiality*

|  | Five Features of Referentiality | | | | |
| --- | --- | --- | --- | --- | --- |
| Potential Referential Gesture | 1. Directed Towards an Object | 2. Directed Towards a Potential Recipient | 3. Receive a Voluntary Response | 4. Are Mechanically Ineffective | 5. Hallmarks of Intentionality |
| Roll over | Y | Y | Y | Y | Y |
| Lean back | N | N | N | N | Y |
| Head forward | Y | Y | Y | Y | Y |
| Head back | N | N | Y | N | Y |
| Nose | Y | Y | Y | Y | Y |
| Body Lean | N | Y | Y | N | Y |
| Shuffle | Y | Y | Y | Y | Y |
| Paw | Y | Y | Y | Y | Y |
| Lean forward | N | Y | Y | N | Y |
| Back leg up | Y | Y | Y | Y | Y |
| Paw hover | Y | Y | Y | Y | Y |
| Chin rest | Y | Y | N | N | Y |
| Groaning | N | N | N | N | Y |
| Lick | Y | Y | Y | Y | Y |
| Paw rest | Y | Y | Y | Y | Y |
| Puppy dog | N | Y | Y | N | Y |
| Front paws on | Y | Y | Y | Y | Y |
| Chomp | Y | Y | Y | Y | Y |
| Head turn | Y | Y | Y | Y | Y |
| Head rest | Y | N | N | Y | Y |
| Head rub | Y | Y | Y | Y | Y |
| Lick lips | N | N | N | N | Y |
| Hind leg stand | Y | Y | Y | Y | Y |
| Head up | Y | N | N | Y | Y |
| Stamp paws | N | N | N | Y | Y |
| Begging gesture | N | Y | Y | Y | Y |
| Turn | N | N | N | N | Y |
| Grab toy | Y | N | N | N | Y |
| Flick toy | Y | Y | Y | Y | Y |
| Rest & wait | Y | N | N | N | Y |
| Drop toy | Y | N | N | N | Y |
| Circle | N | N | N | Y | Y |
| Hover & wait | N | N | N | Y | Y |
| Look behind | N | Y | Y | N | Y |
| Sway | N | N | N | N | Y |
| Paw shove | Y | Y | N | Y | Y |
| Spin bounce | N | N | Y | N | Y |
| Jump | Y | Y | Y | Y | Y |
| Side-step | N | N | N | N | Y |
| Toy in mouth | Y | N | N | N | Y |
| Rub head | N | Y | Y | Y | Y |
| Down-up | N | Y | Y | N | Y |
| Knock on door | Y | N | N | N | Y |
| Crawl under | Y | Y | Y | Y | Y |
| Paw reach | Y | Y | Y | Y | Y |
| Head down | N | N | N | N | Y |
| Head under | Y | Y | Y | Y | Y |

*SM4.* *Referential gestures observed in each subject during the four ASOs.*

| Subject ID | Referential Gestures in ASOs | | | |
| --- | --- | --- | --- | --- |
|  | ***1. “Scratch me!”*** | ***2. “Give me food/drink”*** | ***3. “Open the door”*** | ***4. “Get my toy/bone”*** |
| To.S | Roll over | Paw | Nose; Head turn |  |
| Pu.S |  | Head turn |  | Head under; Paw |
| Ma.W | Head forward; Front paws on; Head turn; Paw;  Lick | Head turn; Hind leg stand; Nose; Front paws on; Jump; Head forward | Head turn; Lick; Front paws on |  |
| Sh.L | Nose; Roll over; Lick |  | Paw; Jump; Front paws on; Lick; Hind leg stand |  |
| Wa.L | Roll over; Jump | Head turn; Head forward | Head turn; Jump; Front paws on; Head forward |  |
| St.W | Roll over; Shuffle | Head turn; Nose; Paw; Jump; Lick; Front paws on; Head forward; Hind leg stand | Head turn; Nose; Lick | Head under; Paw reach; Head turn |
| Ma.Wd | Nose; Paw; Head forward | Head turn | Jump |  |
| Su.W | Roll over; Back leg up; Nose; Head turn; Lick | Head turn; Head forward |  |  |
| Pa.B | Roll over | Head turn | Head turn; Nose; Front paws on; Jump | Paw reach; Head under; Jump |
| Em.P | Paw hover; Head forward; Head turn | Head turn; Head forward | Head turn | Head under; Paw |
| Ti.D |  | Head turn; Paw hover | Head turn; Paw hover; Front paws on |  |
| Te.D |  | Head turn | Head turn; Lick; Paw hover; Head forward |  |
| De.F | Nose; Lick | Head turn; Hind leg stand | Head turn | Paw reach; Head turn |
| Lo.S | Roll over; Paw; Nose; Head turn | Paw; Lick; Head turn; Front paws on; Jump; Lick; Paw hover | Jump; Front paws on; Head turn | Nose; Head under; Head turn; Front paws on; Hind leg stand; Paw |
| Ma.B | Nose; Paw; Head turn | Head turn; Paw; Nose; Front paws on; Head forward; Lick | Head turn; Paw | Head under; Head turn; Crawl under; Paw; Paw hover |
| Ph.J |  | Head turn; Front paws on; Jump | Head turn; Front paws on; Paw; Jump; Head forward | Paw reach; Head under; Paw; Nose; Paw hover |
| Fl.H | Paw; Lick; Nose; Head turn | Head turn; Jump; Front paws on | Head turn; Paw; Paw hover | Head under; Head turn; Nose |
| Os.B |  | Flick toy; Hind leg stand; Head turn; Front paws on; Paw hover; Head forward | Head turn; Paw | Head turn; Head under; Paw reach |
| Ky.H |  | Head turn |  | Head under; Nose; Paw reach; Paw; Head turn |
| Pe.S |  | Head turn | Head turn; Front paws on; Head under | Head turn; Lick |
| Mi.C | Paw; Nose; Lick; Paw hover; Head turn | Lick; Head turn; Flick toy | Head turn; Front paws on; Paw; Nose |  |
| Ru.S |  | Head turn; Paw;  Nose; Front paws on; Jump |  | Head under; Head turn; Paw; Nose; Paw reach |
| Aa.S |  | Head turn |  |  |
| Iz.W |  | Head turn; Paw; Nose; Jump | Head turn |  |
| Ly.W | Lick; Front paws on | Head turn; Hind leg stand; Lick; Front paws on | Head turn |  |
| Le.T |  | Head turn; Front paws on; Head forward | Head turn; Jump |  |
| Yo.B | Lick; Nose; Roll over; Paw hover; Head turn; Front paws on; Head forward | Head turn | Head turn |  |
| Bo.H | Head turn; Nose | Head turn; Paw hover | Paw; Nose; Head turn | Paw; Head turn; Paw hover |
| Du.L | Head turn |  | Head turn |  |
| Bo.L |  | Nose; Head turn |  |  |
| Ar.H |  |  |  | Head under |
| Ja.B | Nose; Lick | Head turn | Head turn; Nose; Paw | Head under; Head turn; Nose; Paw |
| Je.S | Lick; Chomp; Paw; Nose; Head turn |  | Front paws on;  Head turn |  |
| Je.K | Paw hover; Paw; Front paws on | Head turn; Paw; Paw hover |  |  |
| Ba.K |  | Head turn |  |  |
| Be.C | Roll over; Head turn | Head turn; Paw; Lick | Head turn |  |
| On.C | Head rub | Head turn; Paw; Front paws on | Paw; Nose |  |
